# Removal of Part of the Base of the Tongue

**Published:** 1845-01

**Authors:** 


***Removal of part of the Base of the Tongue.***—M. Sédillot operated in the following manner in a case in which the left half of the tongue was involved in cancerous ulceration, extending back, nearly to the epiglottis. After extracting the first left incisor tooth, a vertical incision was made to the left of the median line, through the lower lip, the integuments of the chin and neck, down to the os hyoides. A narrow bistoury was then passed behind the corresponding portion of the maxilla, after which the bone was divided by a single stroke of the saw. Two assistants having then separated the branches of the maxilla, the soft parts of the left side were divided as far back as the palate by means of a straight bistoury, and then the diseased portion of the tongue was removed by an incision through the mesian line, carried out behind with a sweep, parallel with the epiglottis. The lingual artery was tied: the dressing consisted in placing the portions of the maxilla in apposition, and so retaining them by means of a plate of gold, retained in front of the teeth by means of a silk thread. The lip was brought together by means of the twisted suture, and an opening was left in the integuments of the neck, for the passage of pus and mucus. In nine days after the operation, the lip became united, the jaw-bone consolidated, the wounds of the tongue and mouth healed, and everything in fact indicated complete success. There was no tendency to retraction of the tongue, as the *genio-glossus* muscle was uninjured.—*London Med. Times.*

